# In vivo animal study and clinical outcomes of autologous atelocollagen-induced chondrogenesis for osteochondral lesion treatment

**DOI:** 10.1186/s13018-015-0212-x

**Published:** 2015-05-28

**Authors:** Jinsu Kim, Hunki Cho, Kiwon Young, Jaehyun Park, Junkeun Lee, Dongsam Suh

**Affiliations:** Department of Foot and Ankle Surgery, Eulji General Hospital, College of Medicine, Eulji University, 14 Hangeulbiseok-Gil, Nowon-gu, Seoul 139-711 South Korea; RMS, SewonCellontech, Seongdong-gu, Seoul South Korea; 8 F Wooyoung Techno Center, 273-15, Seongsu 2ga 3-dong, Seongdong-gu, Seoul South Korea

**Keywords:** Talus, Cartilage disease, Subchondral arthroplasty, Atelocollagen, CartiFil

## Abstract

**Background:**

Collagen acts as a scaffold for healing damaged cartilage. This study evaluated the results of an in vivo animal study and provides short-term clinical results on a mixture of atelocollagen and fibrin glue-enhanced microfracture techniques in patients with osteochondral lesions (OCL) of the talus.

**Methods:**

This paper contains animal in vivo data and clinical outcomes on the effectiveness of atelocollagen. An in vivo animal study was conducted with full-thickness cartilage defects created in the femoral condyle of 12 rabbits equally divided into 4 groups evaluated at 2, 4, 8, and 12 weeks. Four chondral lesions were created according to one procedure on each rabbit with each lesion treated as follows: (1) microfracture, (2) microfracture and the lesion covered with atelocollagen, (3) microfracture and the lesion covered with mixture of atelocollagen and fibrin glue, and (4) microfracture and the lesion covered with fibrin glue. In the clinical evaluation, 17 patients were treated with a combination of microfracture and atelocollagen injection for symptomatic full-thickness OCL of the talus. They were evaluated by the American Orthopedic Foot and Ankle Society Ankle-Hindfoot Score (AOFAS), Hannover Ankle Score System (HSS), visual analog scale (VAS), and magnetic resonance imaging (MRI) at baseline and at 12-months follow-up. Magnetic Resonance Observation of Cartilage Repair Tissue (MOCART) score of the post-op status was compared with the MOCART score and a modified Anderson’s score of the pre-op status.

**Results:**

In the animal study, subchondral bone and cartilage were generated completely in groups 2 and 3 microscopically. Hyaline-like cartilage was found in the repair tissue. In the clinical evaluation, mean AOFAS improved from 62 to 88, mean HSS improved from 62 to 87, and mean VAS score improved from 64 to 18, respectively (*p* <0.001). Fifteen patients (89 %) reported good or excellent satisfaction. We defined the improvement of most of the subchondral bone edema and bone cyst as well as a chondral lesion by radiologic evaluation.

**Conclusions:**

Rapid regeneration of cartilage was demonstrated in the in vivo animal study, and patients showed significant clinical improvement. Atelocollagen-enhanced microfracture enabled a reasonable treatment of cartilage defects.

## Background

Traumatic and degenerative cartilage injury occurs frequently in knee and ankle joints; however osteochondral lesions receive more attention due to their correlation with the progression of joint degeneration [1]. Adult cartilage has very low intrinsic activity and low potential for regeneration. Cell migration does not occur due to low vascularity and high stiffness. Consequently, the histological characteristics of cartilage that cannot form cell clumps lead to a lack of essential cells and collagen that is unable to recruit local sources of progenitor cells at the articular surface as well as the synovial lining of the joint cavity [2]. Therefore, healing of chondral and osteochondral defects may not be expected. Microfracture, osteochondral autologous transplantation (OATS), and ACI are the most well-known treatments for these injuries [3]. Especially, microfracture is widely used and induces the repair of localized articular cartilage defects of the talus with encouraging mid-term and long-term follow-up results [4, 5]. However, microfracture has several limitations. First, fibrin clot is not mechanically stable enough to withstand tangential forces [6] and is also easily washed out by synovial fluid. Second, they are damaged by axial forces, and the regeneration abilities of the chondrocytes may disappear. Last, damaged parts are filled with fibrous cartilage instead of hyaline-like cartilage. Fibrin glue and biomembrane, types of collagen, were developed as a scaffold due to the slight advantage of microfracture and for compensating stability. As described by Gille et al., an implanted exogenous scaffold (e.g., collagen matrix) may improve mechanical stability; in addition, the durability of cellular environment proved beneficial for the chondrogenic differentiation and cartilage regeneration [7, 8].

Collagen is often used as a scaffold during cartilage regeneration. Collagen-gels have been used for in vitro and in vivo cartilage-regeneration experiments [9, 10], with some applied to clinical cases [11, 12]. CartiFill™ (atelocollagen, Sewon Cellontech, Seoul, Korea) is an atelocollagen that satisfies the scaffold criteria and represents an initial structural protein made from osteocytes during the bony healing process. Atelocollagen exhibits an ideal biocompatibility due to reduced antigenicity from the removal of telopeptide and the structural similarity of porcine collagen to human collagen versus bovine collagen. It also aids in the regenerative process through various tissue-specific biological substrates and helps the fibro-cartilaginous complex to fix chondral defect sites more firmly.

We hypothesize that atelocollagen helps regenerate the osteochondral defect that acts as a scaffold for the in vivo animal study and clinical outcomes. This study is a macroscopic observation of the regeneration of articular cartilage in an in vivo animal study; in addition, it shows the efficacy of atelocollagen through an evaluation of clinical and radiologic outcomes in preliminary clinical studies.

## Methods

**Fig. 1:**
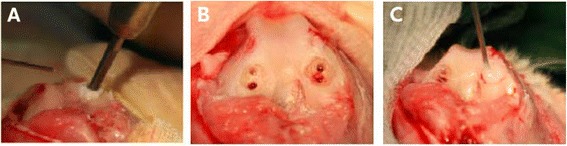
In vivo animal study procedure. Microfracture done at the femoral condyle cartilage of a rabbit, and the lesion was covered with scaffolds. **a** Microfracture done at the femoral condylar cartilage lesion of a rabbit. **b** Result of microfracture (chondral lesion, diameter, 4 mm; depth, 3 mm) **c** covered with scaffold material (fibrin glue or atelocollagen or mixture of fibrin glue and atelocollagen)

All animal experiments were conducted according to guidelines provided by the animal committee of the central government (SC-IACUC-11-004). We compared the effects of microfracture and atelocollagen-enhanced microfracture in rabbits. Twelve mature male New Zealand white rabbits (weight, 3.5–4.5 kg) were divided into four groups for evaluation at 2, 4, 8, and 12 weeks. All surgeries were performed under sterile conditions. Each animal was premedicated according to weight with an intramuscular injection of Zoletil (Virbac, Carros, France) and xylazine (Bayer, Leverkusen, Germany) to obtain effective anesthesia. A 3-cm medial parapatellar incision was made over the knee, and the patellar was everted. A full-thickness defect (4 mm in diameter, 3 mm in depth) in the articular cartilage and subchondral bone was made with an electric burr applied to the femoral condyle of the femur. Guidelines of the American Society for Testing and Materials (ASTM) International indicate that the critical size of a chondral defect in rabbit is 3 mm in diameter [13]. Consequently, this study made four bilateral full-thickness cylindrical osteochondral defects (4 mm in diameter). All defects used Kirschner-wire to induce a microfracture before the following four procedures: (1) no treatment for control, (2) microfracture and covering of the lesion with atelocollagen, (3) microfracture and covering of the lesion with a mixture of atelocollagen and fibrin glue, and (4) microfracture and covering of the lesion with fibrin glue (Fig. 1). Implants were then inserted into the defect, and after reducing the patella, the capsule and muscle were closed with a double 3–0 VICRYL (Ethicon Inc., Somerville, USA) suture, and the skin was closed with a continuous 3–0 Nylon (AILEE, Pusan, South Korea) suture. After the operation, a splint immobilized the knee joint for 10 days. Rabbits were maintained in small cages for 2 weeks and allowed to move freely. Knee joints were harvested at 2, 4, 8, and 12 weeks after the operation and macroscopically assessed for surface regularity, surface area, and regenerated tissue color. Harvested knee joints were simultaneously fixed in 10 % phosphate-buffered formalin for at least 24 h at room temperature. After fixation, specimens were decalcified, dehydrated, cleansed, and embedded in paraffin. The 5-mm-thick sections of embedded tissues were cut using a microtome and stained with Hematoxylin-Eosin. Immunohistochemistry with antibodies for type-II collagen was performed according to a standard ABC protocol (Vector Laboratories, Burlingame, USA). The type-II antibody was produced in-house. Specimens were graded with a modified histological scale described by Wakitani et al*.* [14], Pineda et al. [15], and O’Driscoll et al. [16]. The scale had seven categories that assigned a score of 0 to 23 points (lower scores represent superior cartilage regeneration). The clinical evaluation used a retrospective analysis of prospectively collected clinical data from 17 patients (10 males and 7 females) treated with a combination of microfracture and covering with a mixture of atelocollagen and fibrin glue that was evaluated and approved by the institutional review board (EMC IRB09-08) prior to the procedure. From 1 May 2011 to 31 April 2011, we identified 52 patients with osteochondral lesion of talus (OLT) who required surgical treatment due to the failure of conservative treatments; subsequently, we included 17 patients who agreed with informed consent in a preliminary clinical trial. The study included patients with osteochondral lesions of the talus (OLT) who were in pain or had limitations of function despite 6 months of conservative treatment like physical therapy (hot pack, ultrasound and transcutaneous electrical nerve stimulation), medication (NSAIDs), and hyaluronic acid injection every week for three times. Patients also had protected weight-bearing using orthosis and strengthening exercises when ankle instability was observed. All patients were primary cases and were asked about general health information to exclude patients who had diseases that could affect the healing process of the talus, including rheumatic diseases, systematic inflammatory disease, crystal-induced arthropathy, or diffuse arthritis with at least Kellgren lawrence grade II or TAKAKURA grade II. All lesions were checked with plain X-rays and magnetic resonance imaging (MRI) to measure size and diagnose associated pathology. Preoperative MRI was evaluated with a modified Anderson’s score [17]. A marked probe measured the lesions during the operation. We assume that collagen defects are elliptical and that the defect size was calculated (multiplication of coronal and sagittal length and 0.79). A surgeon diagnosed other ankle diseases such as instability or Os subfibulare by physical examination and a radiological evaluation. Chronic ankle instability and Os subfibulare occurred in ten and two cases, respectively. A modified Bröstrom procedure (a lateral ligament augmentation procedure with superior extensor retinaculum) was conducted in four patients, arthroscopic anterior talofibular ligament (ATFL) reconstruction was done in six patients, and excision of Os subfibulare was done in two patients, with plate and screw removal done in one patient.

Clinical outcomes were measured with the following: American Foot and Ankle Society Ankle-Hindfoot Score (AOFAS), the Hannover Ankle Score System (HSS), and a visual analog scale (VAS) at baseline and at 12 months. Patient satisfaction with outcomes was scored by four grades (excellent/good/fair/poor). A post-operative follow-up MRI was done at 12 months and classified according to a Modified Magnetic Resonance Observation of Cartilage Repair Tissue (MOCART) score [18] and a modified Anderson’s score.

**Fig. 2:**
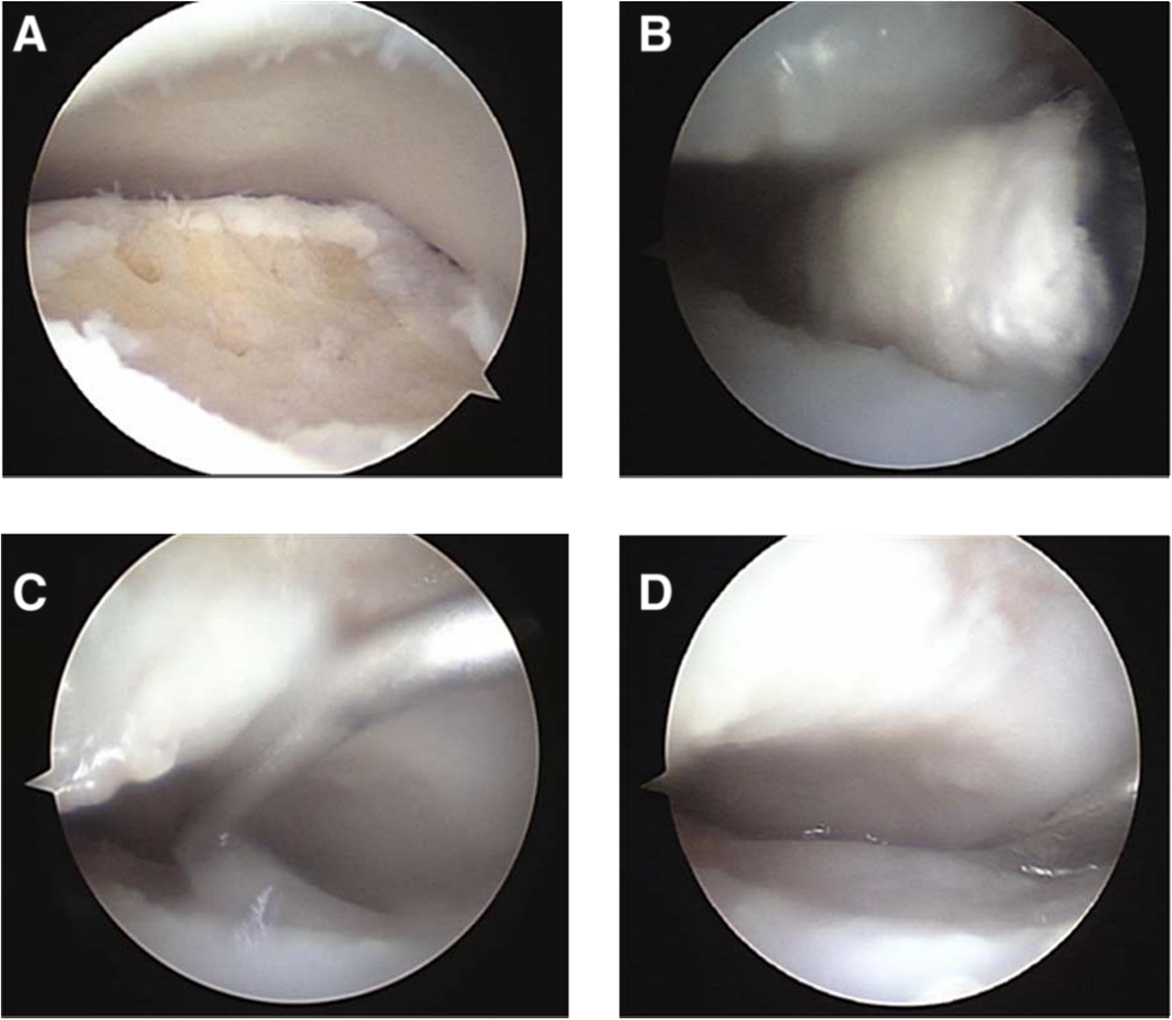
Surgical procedure. **a** Microfracture was done. **b** Removal of the intra-articular fluid by suction and prepared talus as dry status by Endo Peanut. **c** Injection of the mixture of atelocollagen and fibrin glue as a slow drip. **d** A 5-min wait to harden the substances

All procedures were performed under general anesthesia by a single experienced surgeon using an arthroscopic technique with tourniquet. All lesions were accessible using two standard anteromedial and anterolateral portals. A blunt trocar with a 30° arthroscope was inserted through the anteromedial portal into the joint. Arthroscopic synovectomy was done along with a careful examination of the status of the talar dome. Unstable chondral fragments were completely excised with a curette and shaver; additional debridement was performed if the subchondral bone was weak. Fat droplets were checked after microfractures were placed 4–5 mm apart and 4–5 mm deep. The ankle joint was suction dried to remove intra-articular fluid. A two-way syringe injected the mixtures through the portal with a slow injection procedure done under arthroscopic sight to prevent overflow. The procedure made several atelocollagen layers and repeatedly attaching layers on the prior atelocollagen layer to form a complete seal. We waited 5 min for the atelocollagen to harden and then closed the wound in layers, approximated the skin with absorbable subcutaneous sutures, and applied a compressive dressing (Fig. 2). Patients were splinted for 4 weeks before a range of motion exercises was started with a brace. Partial weight bearing was encouraged at 2 weeks postoperatively, and full weight bearing was permitted at 4 weeks. Patients were allowed to jog 3 months after the operation and return to normal sports activity at 6 months follow-up.

### Statistical analysis

Differences between preoperative and post-operative AOFAS, VAS, and HSS were determined using a paired t-test. A Pearson’s correlation test statistically evaluated the postoperative clinical score (AOFAS score, Hannover score, and VAS score) and MOCART score. A *p* value <0.05 was considered statistically significant (SPSS 16.0 for Windows, Chicago, Illinois).

## Results

### In vivo animal study

**Fig. 3:**
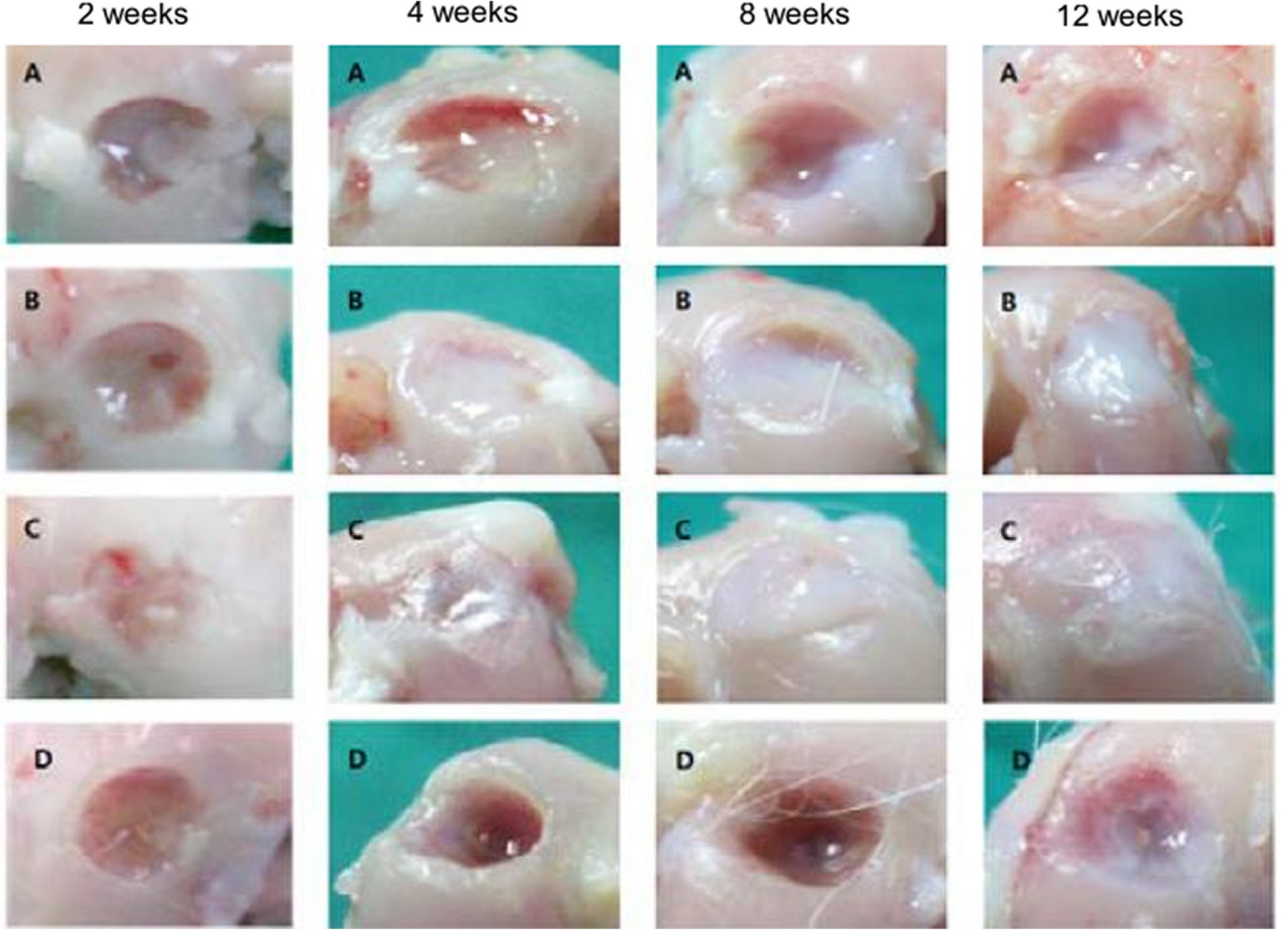
Macroscopic view of the repaired tissue after 2, 4, 8, and 12 weeks after implantation in the in vivo study. **a** Microfracture. **b** Microfracture + atelocollagen. **c** Microfracture + atelocollagen + fibrin glue. **d** Microfracture + fibrin glue 2, 4, 8, and 12 weeks

**Fig. 4:**
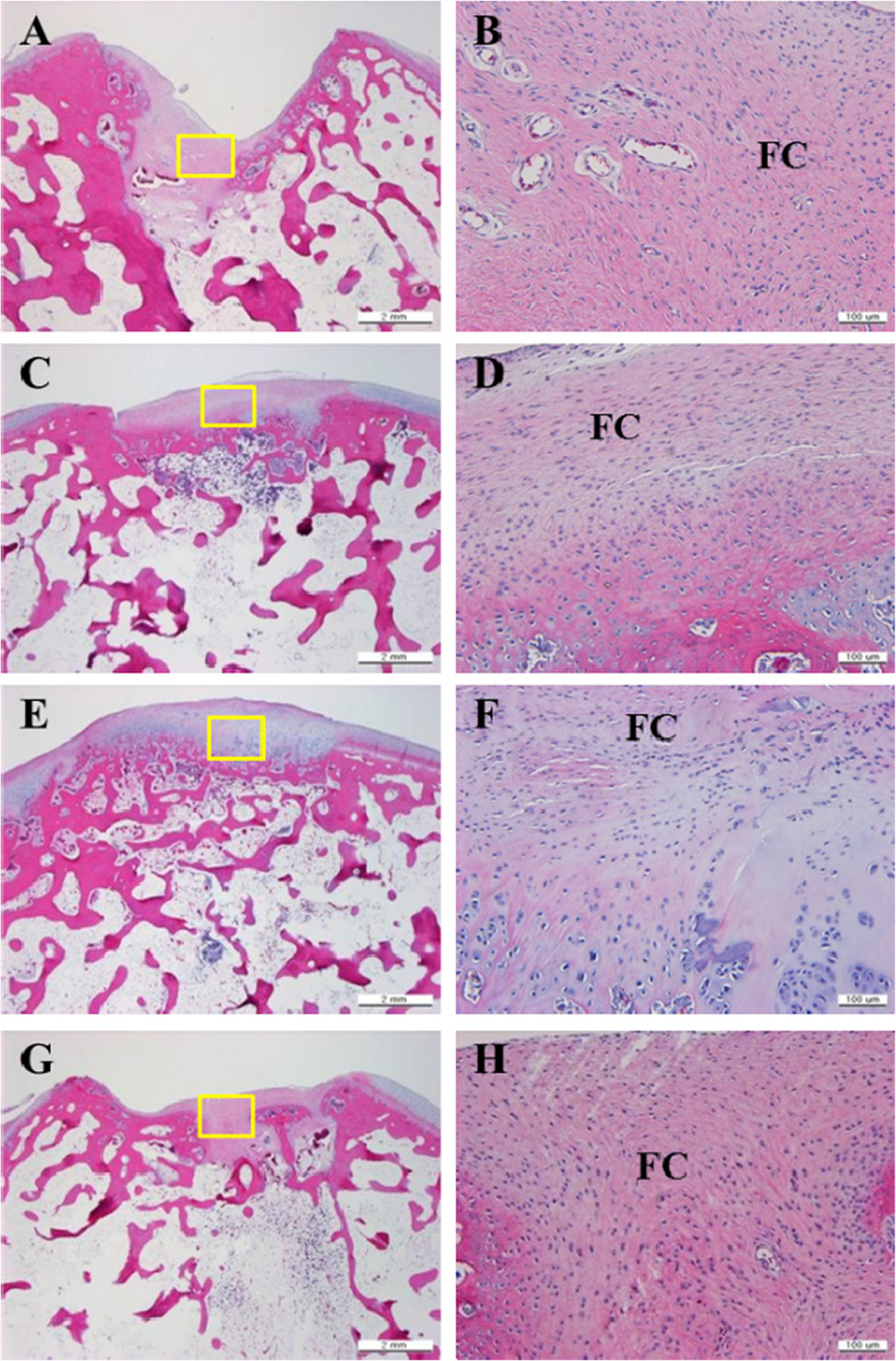
Microscopic view of repaired tissue 12 weeks after operation (hematoxylin/eosin). Cartilage defects in groups 1 and 4 were not adequately filled with repaired tissue. Fibrocartilage tissue is also found in repaired tissue instead of normal hyaline cartilage. Defect completely filled with repaired tissue in groups 2 and 3, and composed of normal hyaline cartilage. **a**, **b** Group 1. **c**, **d** Group 2. **e**, **f** Group 3. **g**, **h** Group 4. *right panel is a magnified view of the *yellow box* in the left panel. *HC* hyaline cartilage, *FC* fibrocartilage

**Fig. 5:**
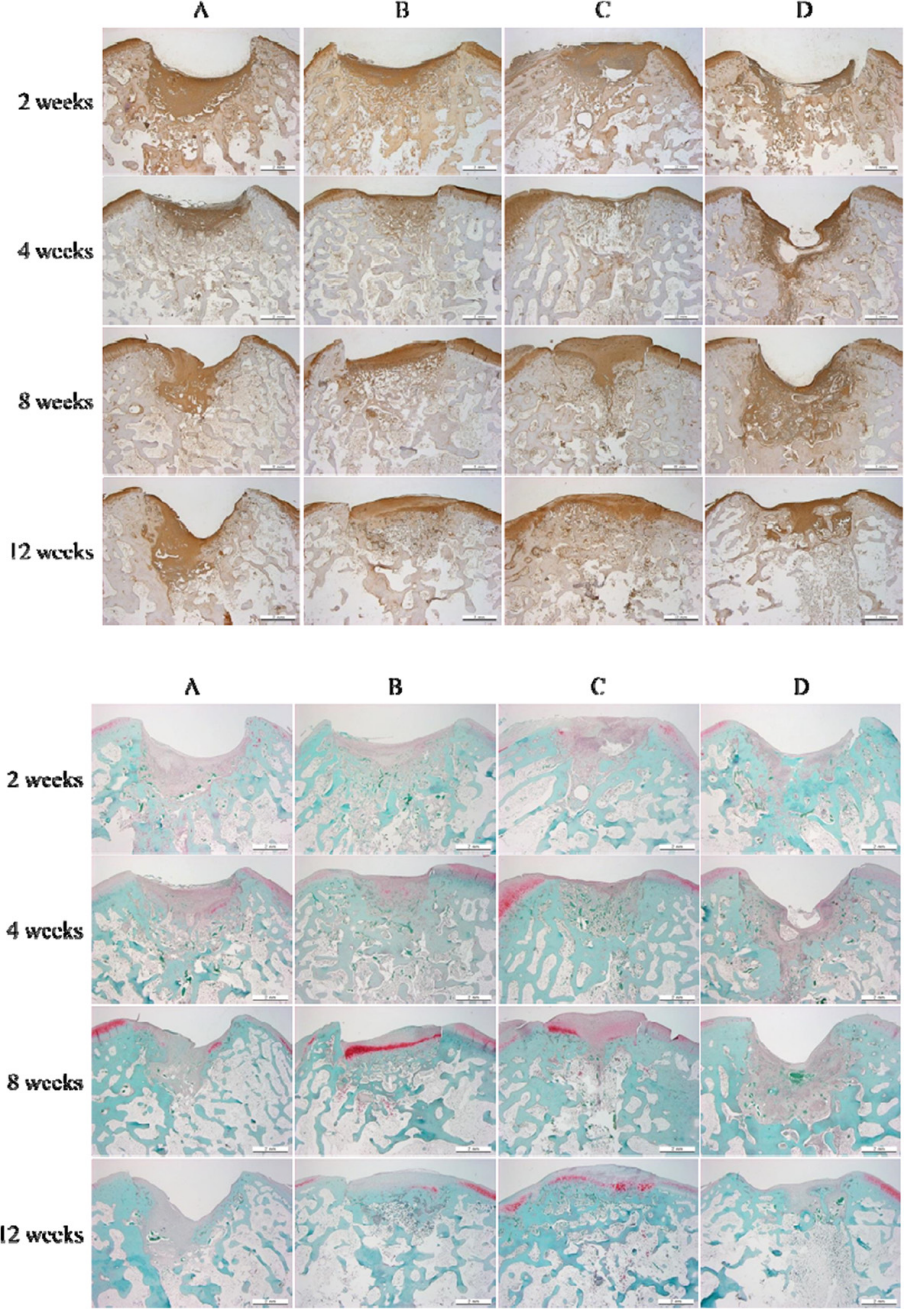
Microscopic view of repaired tissue 12 weeks postoperatively (collagen type-II stain, safranin O stain). **a** Microfracture. **b** Microfracture + atelocollagen. **c** Microfracture + atelocollagen + fibrin glue. **d** Microfracture + fibrin glue

Most lesions in group 3 were macroscopically filled with repair tissue at 4 weeks after operation. At 12 weeks, lesions were completely filled with regenerated tissue in groups 2 and 3; however, the defect was still caved without repaired tissue in groups 1 and 4 (Fig. 3). A microscopic view indicated that the cartilage defects were sufficiently filled with repair tissue; in addition, regeneration of subchondral bone was not detected 12 weeks post operation in groups 1 and 4. Subchondral bone and cartilage were almost generated completely in groups 2 and 3. Group 3 indicated subchondral bone and cartilage repair that smoothly linked with adjacent normal cartilage. Group 2 indicated subchondral bone repair partially that connected with normal cartilage on only one side of the defect (Fig. 4). The regenerated tissues in groups 2 and 3 also reacted strongly with the collagen type-II stain and safranin O stain. The collagen type-II stain showed that the regenerated tissues were hyaline-like cartilage which is histologically the same as normal cartilage (Fig. 5). Table 1 shows the histological scoring for in vivo animal study. At 12 weeks, groups with atelocollagen significantly improved compared to the fibrin glue group; subsequently, the group with a mixture of atelocollagen and fibrin glue showed the best results.

**Table 1:** Histological scoring for in vivo rabbit study

	Histological scoring (mean ± SD)			
	Control	Fibrin glue	3 % atelocollagen	Mixture of fibrin glue and atelocollagen
2 weeks	21.3 ± 0.6	20.3 ± 0.6	20.0 ± 1.0	17.7 ± 1.5^a,b^
4 weeks	18.3 ± 0.6	20.0 ± 1.0	15.7 ± 2.1^b^	13.7 ± 0.6^a,b^
8 weeks	18.7 ± 0.6	19.7 ± 0.6	14.0 ± 1.0^a,b^	11.0 ± 1.0^a,b,c^
12 weeks	19.0 ± 2.0	14.7 ± 1.5^a^	12.0 ± 1.0^a^	9.7 ± 2.3^a,b^

*n* = 3

^a^ *P* <0.05 compared to the control group at each week

^b^ *P* <0.05 compared to the fibrin glue group at each week

^c^ *P* <0.05 compared to the 3 % atelocollagen group at each week

### Clinical outcome

Seventeen patients were treated with a mixture of atelocollagen and fibrin glue-enhanced microfracture (10 males and 7 females). The mean follow-up was 16 ± 4.2 months. No patient was lost to follow-up to check clinical outcome; however, six patients declined to be evaluated by MRI. Mean defect size was 71.9 ± 36.0 mm^2^ (range, 9.4–156.4 mm^2^) (Table 2). The clinical outcome of all patients improved, and the average AOFAS score was 62 ± 12.2 (range, 36–80) before surgery and 88 ± 6.7 (range, 75–100) 12 months after implantation. The average VAS was 64 ± 12.4 mm (range, 40–85) at screening and 18 ± 7.9 mm (range, 5–35) 12 months after implantation. The average preoperative HSS score was 62 ± 9.1 (range, 51–77) and increased to 87 ± 8.7 (range, 73–96) at the 12 month visit. The AOFAS score, VAS, and HSS score improved significantly 12 months after implantation (*p* < 0.001). Patient satisfaction numbers were nine excellent (53 %), six good (36 %), two fair (11 %), and none poor (0 %) at the 12-month visit. A total of 15/17 patients (89 %) scored satisfaction with the technique as good. Table 3 illustrates the radiographic score of patients at pre-operation and follow-up. Table 4 records the modified Anderson’s score for the cartilage condition of 11 patients who got the MRI evaluation before and after operation. Their results indicate an overall improvement in cartilage condition. Two patients who received arthroscopy complained of transient numbness of the skin that was innervated by a superficial peroneal nerve but improved with no treatment 1 month after the operation. Subgroups of patients were statistically evaluated to determine the influence of the operation on postoperative results; however, no significant correlations were found between age, body mass index (BMI), size, bone cyst, edema, sex, ankle lateral instability, OSF lesion, clinical score, or MOCART score.

**Table 2:** Demographic data of patients receiving operation for enhanced microfracture of the talus

List	No.
Male/female	10/7
Average patient age (years)	32.9 ± 12.8
Average BMI	25.2 ± 3.1 (24.9 ± 3.5/25.6 ± 2.9)
Average height (cm)	168 ± 6.8 (171 ± 4.5/163 ± 7.0)
Average weight (kg)	71.1 ± 10.2 (73.2 ± 22.8/68.1 ± 9.7)
Average size of osteochondral lesion of talus	71.89 mm^2^ ± 35.0
Previous operative history	1 (lateral malleolar fracture)
Combined ankle problem	
Chronic lateral ankle instability	10
Os foot subfibulare	2
Syndesmosis injury	1
S/P LM fracture (implant removal)	1
Location of osteochondral lesion of talus	
Anteromedial	1
Medial	3
Posteromedial	11
Posterolateral	2
Subchondral bone cyst	3
Subchondral signal increase	5
History of trauma	1

*BMI* body mass index, *S/P LM* status of postoperative lat. malleolar fracture

**Table 3:** Demographic finding of MRI

No.	Initial modified Anderson’s score	Follow-up modified Anderson’s score	Follow-up MOCART score
1	IIA		
2	III	IIC	25
3	IIC		
4	IIB		
5	IIB	IIB	45
6	IIB	IIA	40
7	IV		
8	III	IIA	35
9	III	I	40
10	IV	IIA	45
11	IV		
12	IV	I	35
13	III		
14	I	I	45
15	III	IIB	40
16	IIB	I	45
17	I	0	50

*MRI* magnetic resonance imaging

**Table 4:** Comparison of modified Anderson’s MRI-based classification score between preoperative state and 12-month visit outcome

Stage	Characteristics	Initial MRI	Follow-up MRI (12-month visit)
Stage 0	Normal	0 (0 %)	1 (9.1 %)
Stage I	Marrow oedema	2 (18.2 %)	4 (36.4 %)
Stage IIA	Irregular subchondral bone plate	0 (0 %)	3 (27.3 %)
Stage IIB	Formation of subchondral cyst	3 (27.3 %)	2 (18.2 %)
Stage IIC	Incomplete separation of fragment	0 (0 %)	1 (9.1 %)
Stage III	Unattached, undisplaced fragment with synovial fluid around fragment	4 (36.4 %)	0 (0 %)
Stage IV	Displaced fragment	2 (18.2 %)	0 (0 %)
Total number		11	11

*MRI* magnetic resonance imaging

On the preoperative MRI, three ankles (17.6 %) had bone cysts, and four ankles (23.5 %) had an increased signal of subchondral bone. Two of three ankles with a bone cyst were completely healed and one became 1/5 smaller than the preoperative size. Three of four ankles with an edema of the subchondral bone were significantly reduced; however, a new bone cyst developed from one of them.

## Discussion

The most important finding of the study is verifying the effectiveness of atelocollagen and the mixture of atelocollagen and fibrin glue through an in vivo animal study and the clinical outcome of an operation. Our hypothesis that atelocollagen helps the regeneration of osteochondral defect by acting as a scaffold held true. The result of histology in animal studies indicates that atelocollagen acts as a scaffold at the defect and enhances tissue regeneration by allowing bone marrow cells from the microfracture and surrounding bone (as well as cartilage related cells) to adhere, proliferate, and maturate. Fibrin glue with collagen transform liquid-type collagen to a semi-solid state and can be used as a bioscaffold that provides an increased collagen content, increased numbers of spherically shaped chondrocytes, and elevated mass retention after transplantation [19]. The use of only fibrin glue causes incomplete healing; however, the additional use of atelocollagen optimized healing.

Clinical reports indicate that microfracture is a widely available, simple, and minimally invasive arthroscopic technique associated with good results; however, increasing age, a high BMI, and a large size (lesions >150 mm^2^) produce poor outcomes [20]. Microfracture demonstrates significantly better improvement than autologous chondrocyte implantation for short-term reults; however, a longer follow-up shows that beneficial results are not maintained despite equivalent clinical outcomes that indicate a deterioration after microfracture [3, 21]. Studies have been conducted for large-sized lesions and cases that are predicted to have poor outcomes from microfracture due to microfracture technique flaws such as the instability of clots. To overcome these factors, various enhanced microfracture techniques using scaffolds or progenitor cells have been developed. A mixture of atelocollagen and fibrin glue-enhanced microfracture was recently developed; in addition, its efficacy was reported in only one-knee and ankle-related article, respectively. Ten patients with knee cartilage defects were treated with a similar technique that reported the following: both good clinical and radiologic results at the 2 year follow-up, an MRI with good filling of the cartilage defects, and a biochemical MRI that suggested hyaline-like repair tissue [22]. The difference between our techniques is that we only used suction drying and Endo Peanut without a CO_2_ gas drying process. A recent clinical report about the use of the atelocollagen-fibrin-glue matrix was for the treatment of chondral lesions of the talus, with a significant improvement of VAS and AOFAS score at 6 months follow-up of 5 patients [23]. Our study had two times more patients and a longer follow-up that more accurately evaluated the status of osteochondral lesions by comparing post-operative and pre-operative MRI. An autologous matrix-induced chondrogenesis (AMIC) and other techniques using various kinds of scaffolds already produced satisfactory results for chondral and osteochondral defects of the knee joint prior to ankle [24–26]. However, limited reports have been published on this novel technique, particularly with the ankle compared to the knee joint. First, the AMIC technique has been applied to both chondral and osteochondral lesions of the ankle [27]. A similar novel technique was introduced by Zantop et al. [28]. In our study, all procedures were done easily using an arthroscopic technique different from other techniques performed by open capsulotomy and without donor site morbidity. Different from the AMIC technique, an atelocollagen matrix was mixed with fibrin glue directly and injected easily into the chondral defect.

Increasing age, high BMI, and large lesions are factors that suggest a poor outcome for microfractures [20]; however, we saw no statistically negative effects from these factors in this study. Another focus of ours is on an atelocollagen and fibrin glue-enhanced microfracture that can overcome a simple microfracture; however, the demographics of our population (mean age, 32.9 years and BMI, 25.2) do not fully reflect our focus. Only three patients over 50 years old are included and scored a mean AOFAS of 63.3 in pre-op that later improved to 93.3. Among them, two patients received a post-op MRI and modified Anderson score that improved from IV, IIB to I, I, respectively, with an average MOCART of 40. The superiority of collagen-enhanced microfracture for large osteochondral lesions and elderly patients was not conclusively demonstrated. The biggest lesion in this study was 156 mm^2^, and there were six patients with osteochondral lesions larger than 90 mm^2^. They scored a mean AOFAS of 64 in pre-op that later improved to 86.7. From this result, we expect that further studies with larger groups will confirm that atelocollagen and fibrin glue-enhanced microfracture are effective for elderly patients and large-size osteochondral lesions.

Subchondral bone conditions such as a bone cyst and signal intensity of the repair tissue are concerns. Involvement of the subchondral bone can be seen as sclerotic changes, cyst formation, or avascular necrosis in large talar lesions [29]. The integrity of the subchondral bone plays an important role in the pathogenesis of ankle osteochondral lesions (OCL); however, there is no consensus on the treatment of these defects. Our technique checked bone cysts through pre-op MRI and tried to completely remove differentiated and debrided lesions with a curette. Subchondral bone around the lesion was decorticated and followed by a 6–7 mm-deep microfracture procedure. Good outcomes were obtained without a bone graft; two of three were completely healed and the other bone cyst was 1/5 times smaller but nearly filled with repair tissue. In addition, 75 % (3/4) of patients were cured of subchondral edema, but one patient developed an additional cyst below the microfracture site with edema. The location of the bone cyst indicates it may have been formed by leaked joint fluid from a tiny gap below the bone bed where the microfracture was performed. The clinical result was good with 87 points at AOFAS. We believe that it is suitable to treat bone cysts using our technique without a bone graft; however, more data is needed to generalize the effectiveness of this technique.

Microfracture predominantly produces fibrocartilage [30], and many discussions remain about cartilage made from autologous chondrocyte implantation [31–33]. However, the regenerated cartilage in our in vivo study was a hyaline-like cartilage unlike the subsequent microfracture and ACI. A biopsy could not be performed for ethical reasons; however, good quality cartilage was observed by radiological findings after collagen-enhanced microfracture. Consequently, we expect good results from this procedure.

The current study had several limitations. The clinical study was carried in small scale without a control group to effectively evaluate the efficacy of atelocollagen. An on-going randomized controlled study with power calculation under the same protocols will supplement the lack of data from the previous retrospective study. MRI data weakened by losses in follow-up 35.2 % (6/17) may not represent the whole group; however, most patients did not want further evaluation tests because they were satisfied with a mean AOFAS of 89.2. From this result, we expect the mean MRI evaluation of the whole group to improve. There could also be a minimal selection bias in the evaluation of resolution of pain and disability because there were ten cases of instability and two cases of Os subfibulare as company lesion in the object group. Further mid-term and long-term follow-up must be evaluated. In the in vivo study, a quantitative cell study was not performed in the animal study even though the histologic status of cartilage was evaluated; consequently, additional evaluation is required for a quantifiable comparison. The duplication of human OLT is difficult to make in animals since the intrinsic characteristic and surrounding environment of cartilage are different and may be the cause of different clinical study results in clinical study. Therefore, a larger scale clinical study is necessary to evaluate clinical efficacy.

This study is the first to report an in vivo histologic animal study and the clinical results of radiographic evaluation using MRI when atelocollagen was used for treatment. This study is meaningful in revealing predictable results of atelocollagen for chondral lesion with the number of subjects three times larger and the follow-up period twice as long compared to previous clinical trials. The mixture of atelocollagen and fibrin glue-enhanced microfracture in this experiment is a simple one-step procedure with no need to harvest cartilage, no risk of contamination associated with an in vitro cell culture procedure, and is more cost-effective. This technique also showed a potential to overcome simple microfracture flaws and may be a good option for osteochondral lesion treatment.

## Conclusions

Good results were obtained in macroscopic and histological assessments of the outcomes of an in vivo animal study. Microfracture with a mixture of atelocollagen and fibrin glue was effective and safe to treat OCL of the talus. Patients showed significant clinical improvement and satisfaction postoperatively. Atelocollagen as a one-step procedure enables a satisfactory treatment of cartilage defects.
